# Development of Live-Attenuated Influenza Vaccines against Outbreaks of H5N1 Influenza

**DOI:** 10.3390/v4123589

**Published:** 2012-12-10

**Authors:** Dan Zheng, Yinglei Yi, Ze Chen

**Affiliations:** 1 Shanghai Institute of Biological Products, 1262 YanAn Road(w), 200052, Shanghai, China; E-Mails: daisy_zd@hotmail.com (D.Z.); yiyinglei@163.com (Y.Y.); 2 College of Life Sciences, Hunan Normal University, Changsha Yuelushan 410081, Hunan, China

**Keywords:** influenza, H5N1, live-attenuated influenza vaccines, cross-protection, adjuvants

## Abstract

Several global outbreaks of highly pathogenic avian influenza (HPAI) H5N1 virus have increased the urgency of developing effective and safe vaccines against H5N1. Compared with H5N1 inactivated vaccines used widely, H5N1 live-attenuated influenza vaccines (LAIVs) have advantages in vaccine efficacy, dose-saving formula, long-lasting effect, ease of administration and some cross-protective immunity. Furthermore, H5N1 LAIVs induce both humoral and cellular immune responses, especially including improved IgA production at the mucosa. The current trend of H5N1 LAIVs development is toward cold-adapted, temperature-sensitive or replication-defective vaccines, and moreover, H5N1 LAIVs plus mucosal adjuvants are promising candidates. This review provides an update on the advantages and development of H5N1 live-attenuated influenza vaccines.

## 1. Introduction

The highly pathogenic avian influenza virus (HPAIV) H5N1 causes high mortality (approximately 60%) in humans. On February 24, 2012, the World Health Organization announced that, since 2003, 587 people have been confirmed of HPAIV H5N1 infection and 346 of them have died. Like all influenza A viruses, H5N1 avian influenza virus (AIV) is undergoing continuous evolution. Although human-to-human transmission of this virus has not yet occurred, it is possible that AIV H5N1 could adapt to human hosts through mutation or reassortment. One of the major host range determinants of influenza viruses is the affinity of viral hemagglutinin (HA) protein for its host cell sialic acid receptor. Avian influenza viruses preferentially bind to the α-2,3 sialic acid receptor, while human influenza viruses preferentially bind to the α-2,6 isoform [1]. This receptor preference could, to some extent, explain the barrier for interspecies transmission. However, in theory, only one or two mutations could effectively convert AIV H5N1 to α-2,6 sialic acid receptor preference [2]. Another possibility for AIV H5N1 transmission in human populations is through reassortment with human-adapted virus subtypes, that is, two subtypes of influenza virus could co-infect a cell and swap genetic segments through reassortment [3]. The H1N1 virus that caused the 1918 pandemic is considered as an AIV undergone through adaptive mutations; the H2N2 virus that caused the 1957 pandemic and the H3N2 virus of 1968 are considered to have gained human adaptation through reassortment [4].

For H5N1 virus, there is little pre-existing natural immunity in human populations. If AIV H5N1 gains the capacity for effective and sustained transmission among humans, it could cause a global influenza pandemic with high morbidity and high mortality. Therefore, the continued circulation of H5N1 virus poses a serious threat to public health. Vaccine development is considered a crucial priority for influenza pandemic preparedness. One of the most effective strategies of vaccines is to imitate natural infection to elicit effective, cross-reactive and long-lasting immunity.

Although the traditional trivalent inactivated influenza vaccine (TIV) is currently the most widely used influenza vaccine, the live attenuated influenza vaccine (LAIV) has several advantages over TIV. Intranasal immunization with LAIV elicits immune responses similar to the various immune mechanisms activated by natural infection of wild-type influenza viruses, but without causing the typical signs or symptoms associated with illness. Many studies have demonstrated that intranasal immunization with LAIV could not only elicit influenza virus-specific secretory IgA antibodies and serum IgG antibodies, as well as T-cell responses, but also could provide cross-protection against heterologous influenza viruses [5]. Secretory IgA is involved in defending the upper respiratory tract, while serum IgG protects the lower respiratory tract [6]. LAIV also induces a robust memory response, including the production of chemokines and cytokines involved in T-cell activation and recruitment, which can then clear the virus rapidly [7]. LAIV immunization also induces CD4^+^ and CD8^+^ T-cell responses targeted to conserved epitopes of core proteins, such as matrix protein (M) and nucleoprotein (NP) [8]. Non-neutralizing antibodies (such as anti-M IgG and anti-NP IgG) may help to speed up the clearance of heterologous viruses. Immunity against heterologous viruses is mainly provided by T-cells and non-neutralizing antibodies, which do not prevent infection, but could limit virus replication and greatly lower disease severity and mortality [8,9]. Therefore, compared with TIV, LAIV could elicit a more sustained immune responses and provide protection against both wild-type viruses of the vaccine strain and viruses of different subtypes [7]. As it is impossible to predict which H5N1 strain might cause an influenza pandemic, flu vaccines in stock shall have broad and cross-reactive immunogenicity [10]. In randomized double-blinded clinical trials evaluating LAIV, it was found that the protective effect of LAIV was superior to that of TIV among adults and children [11,12]. LAIV provided better protection than TIV against both antigenically well-matched and antigenically drifted viruses [13]. LAIV induced significantly greater levels of local IgA antibodies in participants than TIV did, which might be one of the reasons why LAIV was more efficient than TIV [14].

For safety issues of LAIV, reverse mutation is of the most concern. The influenza virus replication process has a mismatch rate of about 10^−4^ to 10^−5^ per nucleotide. Studies have confirmed the likelihood of LAIV virus to reverse back to the wild-type strain virus was extremely low, possibly one in 10^20^ replication cycles [15,16]. A study on the currently marketed live influenza virus vaccine, FluMist^®^, showed that the genotype of the vaccine virus was stable after replication in human hosts [17], as the six internal genes from the donor virus gave the vaccine strain stable characteristics of attenuation [15].

## 2. Development of H5N1 LAIV

Using reverse genetics techniques, live attenuated influenza vaccine acquires multiple mutations in its viral genes that produce the cold-adapted, temperature-sensitive and attenuated phenotypes. The cold-adapted vaccine viruses could replicate and grow only when temperature is below 25 °C and stop growth when temperature exceeds 37.8 °C [18]. The temperature-sensitive phenotypes means that viral replication is highly efficient at 33 °C, but becomes ineffective as temperatures reaches 39 °C, *i.e.*, the ratio of plaquing efficiency at 39 °C to that at 33 °C is 10^−3^or less [19]. The attenuated vaccine refers to a vaccine created by reducing the virulence of a pathogen, but still keeping it viable. An overview of studies of H5N1 LAIV reported so far is presented in Table 1.

**Table 1:** Overview of studies on H5N1 live attenuated vaccines reported so far.

Vaccine type		Mode of modification	Stage of research	Vaccinee	Investigator
Temperature-sensitive		Temperature-sensitive influenza vaccine donor strain (A/Guinea Fowl/Hong Kong/WF10/99 (WF10) H9N2)	pre-clinical	poultry and mouse	Daniel R. Perez [20,21] 2007, 2008
Cold-adapted		Cold-adapted influenza vaccine donor strain (A/Ann Arbor/6/60(H2N2)) and deletion of HA cleavage site	phase I	human	Ruth A. Karron [22] 2009
			pre-clinical	mouse	Amorsolo L. Suguitan [23] 2006
			pre-clinical	chicken, mouse and monkey	Shufang Fan [24] 2009
Truncated	NS1 protein	Truncation of NS1 protein, deletion of HA cleavage site, mutation of PB2	pre-clinical	mouse and poultry	John Steel [25] 2009
		Deletion of NS1 open reading frame and HA cleavage site	pre-clinical	chicken, mouse and ferret	Julia Romanova [26] 2009
	M2 protein	Deletion of M2 cytoplasmic tail and HA cleavage site	pre-clinical	mouse	Tokiko Watanabe [27] 2007
Knockout gene		Knockout of PB2 gene	pre-clinical	mouse	Yoshihiro Kawaoka [28,29] 2011, 2012
Computer-aided rational design		Adjustment/redesign of mast coding regions of PB1, NP and HA based on degree of codon-pair deoptimization	pre-clinical	mouse	Steffen Mueller [30] 2010

### 2.1. Reassortment with a Temperature-Sensitive Vaccine Donor Strain

In the 1960s, temperature-sensitive mutants of influenza A virus were obtained by Robert *et al.* using chemical mutagenesis [31]. These temperature-sensitive viruses could effectively replicate between 33 °C and 39 °C, but their replication was restricted at temperatures higher than 39 °C. In 1978, Brian *et al.* reported the result of a clinical trial that the LAIV shed retained its temperature-sensitive phenotype in most instances and failed to spread to susceptible contacts [32]. In 1996, Louise *et al.* found that some mutations in PB1, PB2, PA, M and NS genes might contribute to attenuation in influenza virus [33]. Jin *et al.* demonstrated that influenza A virus containing at least the four loci in the PB1 (K391E, E581G and A661T) and PB2 (N265S) genes exhibited both temperature-sensitive and attenuated phenotypes [34].

By using reverse genetics, Daniel R. Perez and his collaborators modified the internal backbone of A/Guinea Fowl/Hong Kong/WF10/99 (WF10) H9N2 influenza virus. Temperature-sensitive mutations in the PB1 (K391E, E581G and A661T) and PB2 (N265S) were introduced into the WF10 virus backbone, producing a similar temperature-sensitive phenotype. Moreover, an eight-amino-acid hemagglutinin (HA) epitope tag was cloned in frame at the C-terminus of PB1 gene for further attenuated phenotype. Based on the modified WF10 backbone, Perez’s group reconstituted an H5N1 LAIV candidate strain of which the HA (with the deletion of the polybasic amino acid sequence) and N1 genes were from A/VietNam/1203/2004. The temperature-sensitive H5N1 LAIV could provide effective protection for poultry and mice against the HPAI H5N1 virus challenge [20,21]. Moreover, their further research indicated that the modified WF10 backbone could be used to prepare H7N2 and H9N2 LAIVs for *in ovo* vaccination against avian influenza [35]. Due to the outbreak caused by the novel swine-origin influenza (H1N1) in 2009, Perez’s group further demonstrated that the double attenuating mutations implemented for the WF10 virus could be transferred to swine-like influenza viruses (A/turkey/Ohio/313053/04 (H3N2) or A/swine/Wisconsin/14094/99 (H3N2)), and the obtained H3N2 LAIV could induce excellent protection against aggressive H1N1 virus challenges in more than one animal model [36,37]. These results highlighted the availability of the modified WF10 backbone used as a master donor strain for preparing LAIV and its potential for H5N1 LAIV development.

### 2.2. Reassortment with a Cold-Adapted Vaccine Donor Strain

In 1967, by gradually lowering the temperature during serial passage, Hunein Maassab developed a cold-adapted influenza virus (A/Ann Arbor/6/60 (H2N2), AA *ca.*), which could replicate efficiently at 25 °C. Jin and other researchers reported that this cold-adapted strain had five mutations in the PB1 (K391E, E581G, A661T), PB2 (N265S) and NP (D34G) genes [38]. Mutations in these sites rendered the strain capable of effective replication at between 25 °C and 33 °C, but incapable of this activity at higher than 39 °C; these phenotypic characteristics were classified as cold-adapted and temperature-sensitive [39].

FluMist^®^ is the first LAIV (manufactured by MedImmune, Gaithersburg, MD, USA, 2003) with six internal genes of cold-adapted A/Ann Arbor/6/60 or B/Ann Arbor/1/66 as a backbone and also the first nasally administered vaccine on the U.S. market. In 2011, this vaccine was approved by the European Medicines Agency and marketed in the EU under the label Fluenz^®^. FluMist^®^ is a cold-adapted trivalent live-attenuated influenza vaccine manufactured by using reverse genetics techniques. Clinical studies have demonstrated that the vaccine has good safety and efficacy in both adults and children, and it could elicit immune responses in humans to prevent influenza virus infection. Given that H5N1 virus is a severe threat to public health, MedImmune and the U.S. National Institutes of Health (NIH) prepared three H5N1 subtype LAIV candidates whose HA and NA were derived, respectively, from the 1997 Hong Kong, 2003 Hong Kong and 2004 Vietnam H5N1 influenza viruses isolated from humans, while the remaining genomic segments came from the cold-adapted influenza vaccine donor strain (A/Ann Arbor/6/60 (H2N2), AA *ca.*). In addition, for all the above mentioned LAIV candidate strains, their HA proteins have been modified to delete the cleavage site [40]. Deletion of the cleavage site gave these three candidate vaccine strains characteristics of low pathogenicity phenotype, limited replication in respiratory tract of mice and lower pathogenicity in ferrets. Results from an *in vivo* toxicology study in ferrets showed that the candidate vaccine strains had no systemic toxicity after repeated intranasal administration [41]. Pre-clinical studies have demonstrated that the H5N1 LAIV viruses had infectivity, immunogenicity and protective effect in animal models [41]. In June 2006, a phase I clinical study of MedImmune H5N1 LAIVs was conducted by the U.S. NIH. And, in 2009, Karron *et al*. released the results of this clinical study. The cold-adapted H5N1 LAIVs (A/VietNam/1203/2004 and A/Hong Kong/213/2003) bearing avian H5 HA antigens were strictly limited in replication and were more attenuated than seasonal LAIV-bearing human H1, H3 or B HA antigens. Those cold-adapted H5N1 LAIVs could induce serum ELISA antibody, while hemagglutination inhibition antibody and neutralizing antibody could not be detected in healthy adults [22].

In addition, several studies have demonstrated the efficacy of cold-adapted H5N1 LAIVs [23,24]. Suguitan *et al.* conducted a pre-clinical study about safety, immunogenicity and protective effect of a cold-adapted H5N1 LAIV. Due to modification made to the HA gene, the cold-adapted H5N1 LAIV virus could only proliferate effectively in cells when trypsin was added; and as the internal protein genes came from a cold-adapted vaccine donor strain, the vaccine strain possessed the temperature-sensitive and attenuated phenotypes. Intranasal immunization with the vaccine once could protect mice against a lethal challenge of homologous or heterologous wild-type H5N1 viruses; immunization twice could effectively inhibit replication of homologous and heterologous wild-type H5N1 viruses in the lungs [23]. Fan *et al.* prepared a cold-adapted H5N1 LAIV using HA and NA genes of H5N1 A/Anhui/2/05 influenza virus and the backbone of the cold-adapted strain H2N2 A/AnnArbor/6/60 and performed similar experiments in non-human primates [24]. Their results showed that in a non‑human primate model, the H5N1 vaccine could elicit protective immunity against HPAIV H5N1, which provided a convincing argument for further carrying out human clinical studies of H5N1 LAIVs.

### 2.3. Truncation of NS1 Protein or M2 Protein

#### 2.3.1. Truncation of NS1 Protein

The NS1 protein is the non-structural protein encoded by the influenza A virus. It is believed that NS1 protein inhibits the adaptive immune responses via suppressing functions of dendritic cells, and it plays multiple regulatory functions in the virus replication cycle, including regulation of synthesis, transport, splicing and translation of mRNA, which are critical for inhibiting the interferon (IFN)-regulated antiviral response of the host [42]. A virus lacking NS1 protein (delNS1) cannot counter the host IFN response and is highly attenuated in hosts with adequate IFN, but its proliferative ability remains in substrates with IFN secretion deficiency (such as Vero cells) [43].

Based on the H5N1 A/VietNam/1203/2004 virus, Steel *et al.* prepared a series of NS1-truncated LAIV vaccines where the NS1 protein had been truncated to express only the N-terminal 73, 99 or 126 amino acids, respectively; in addition, the strains deleted the cleavage site of the HA gene and mutated at the amino acid 627 of the PB2 protein, thereby further inhibiting their replication capacity. Compared with the wild-type virus, these candidate viruses were highly attenuated in mice. Immunization with the vaccine just once was sufficient to fully protect mice against a lethal dose of homologous viruses. Inoculation of poultry with 10^6^ EID_50_ of the candidate virus (the truncated NS1 protein had 99 amino acids) was sufficient to provide complete protection against a lethal challenge by homologous virus, as well as partial protection against a heterologous H5N1 virus challenge [25]. 

In another study, an NS1-truncated H5N1 LAIV candidate virus was prepared according to a 3:5 gene reassortment, containing three genes of the virus H5N1 A/VietNam/1203/2004 (cleavage site‑deleted HA, NA and M) and five genes from the IVR-116 vaccine virus strain, which had been adapted to grow in Vero cells and modified by the deletion of NS1 open reading frame. In a primate model, intranasal immunization once with the vaccine was enough to elicit antibodies against a challenge by the homologous A/VietNam/1203/2004 or the heterologous A/Indonesia/5/2005 H5N1 virus. A phase I clinical evaluation of the NS1-truncated H5N1 LAIV candidate is currently ongoing [26]. In addition, Wang *et al.* found that A/turkey/Oregon/71-delNS1 (H7N3) virus (10 nucleotide deletion in the coding region of the NS1 gene) could be used as a potential live-attenuated vaccine. The NS1‑truncated H7N3 LAIV candidate viruses were highly attenuated in chickens and did not transmit the virus from infected chickens to uninoculated cage mates. At the same time, the candidate viruses induced relatively high antibody titers, which conferred good protection against a high dose heterologous virus challenge [44].

#### 2.3.2. Truncation of M2 Protein

In virus assembly and pathogenic process, the cytoplasmic tail of M2 has also been demonstrated to play an important role [45]. Based on the basic function of M2 during virus life cycle, Watanabe *et al.* prepared an M2-truncated H5N1 (A/Vietnam/1203/2004) LAIV candidate strain, which deleted the 11 amino acids at the M2 cytoplasmic tail, as well as the HA cleavage site, and, therefore, was referred to as M2del11-HAavir virus. This M2del11-HAavir virus could protect mice against a lethal challenge by homologous (A/Vietnam/1203/2004) or heterologous (A/Indonesia/7/2005) H5N1 virus [27].

### 2.4. Knockout of PB2 Gene

In viral transcription, PB2 protein plays a special role in the process of forming 5'-capped RNA fragments from cellular pre-mRNA molecules [46]. Interestingly, PB2 protein has been found to affect the virulence of influenza viruses [47].

Using reverse genetics techniques, Yoshihiro Kawaoka’s group developed a replication-deficient PB2 knockout (PB2-KO) influenza virus. This recombinant virus was replication defective in wild‑type cells, but it could replicate to high titers in AX4/PB2 cells (stably expressing PB2 protein); it hardly replicated in other cells and, thus, had good safety. Mice immunized with this PB2-KO influenza virus had higher IgG and IgA antibody levels in serum, nasal washes and bronchoalveolar lavage fluid than mice immunized with the traditional inactivated vaccine. When challenged with a lethal dose of influenza virus, all mice immunized with the PB2-KO influenza virus survived. These results confirmed the safety, as well as the feasibility, of the recombinant virus to be used as LAIV [28,29]. 

### 2.5. Computer-Aided Rational Design

Mueller *et al.* applied synthetic attenuated virus engineering (SAVE) to design LAIV vaccine candidates. Attenuation can be ‘titrated’ by adjusting the extent of codon-pair deoptimization. As attenuation is based on changes in hundreds of nucleotides across the viral genome, reversion of the attenuated variant to a virulent form is unlikely. The SAVE technique was used to redesign most of the coding region of PB1, NP and HA genes of influenza virus A/Puerto Rico/8/34. Codon-pair deoptimization proved to have lowered virulence by 13,000-times as compared to the parent strain. Immunization of mice with the codon-pair deoptimized variant by a single intranasal exposure could protect them against a challenge by wild-type influenza virus [30]. This new strategy could be applied to rapid vaccine development, such as H5N1 LAIVs, in response to the threat of an influenza pandemic. 

## 3. Adjuvants Applied in LAIV

Adjuvants act to stimulate the innate immune responses, to enhance antigen presentation and to improve immune responses to vaccination, thereby allowing lowering of the vaccine dose. The safety of LAIV in immunocompromised populations has long been a concern. Application of adjuvant to LAIV could potentially lower the dose of LAIV and increase its safety and allow expansion in populations suitable for vaccination. During a potential future influenza pandemic caused by H5N1 viruses, it is possible that the massive death of infected chickens and subsequent large-scale slaughter of chickens would shrink the supply of chicken embryos at the very time when the demand for vaccine is greatly increased. Among the technical means to solve this problem, using an adjuvant is one way to decrease the amount of vaccines needed. In clinical trials, H5N1 TIV and H5N1 LAIV showed poor immunogenicity [22,48,49]. The use of adjuvant would be a good way to improve the immunogenicity of H5N1 LAIV. Several studies proved that some effective adjuvants could be used in LAIV, including alpha-C-GalCer and chitosan. The available adjuvants for application with LAIV are summarized in Table 2. 

### 3.1. Alpha-C-Galactosylceramide

The adjuvant alpha-galactosylceramide (alpha-GalCer) functions by stimulating natural killer T (NKT)-cells to release cytokines, which in turn activate the adaptive immune response [50]. Alpha-C-galactosylceramide (alpha-C-GalCer), an analogue of alpha-GalCer, has been shown by a recent study to enhance immunostimulatory effects by inducing increased and prolonged production of the Th1‑type cytokines, interferon gamma [51].

**Table 2:** Outlines of studies on adjuvanted LAIV.

Animal model	Mode of immunization	Immunogen	Dose of immunogen	Adjuvant	Dose of Adjuvant	Protection against a homologous challenge	Protection against a heterologous challenge	Investigator
mouse	intranasal	H1N1 LAIV	10^2^ PFU	alpha-C-GalCer	0	100LD_50_ H1N1 (0%)	N. D. ^a^	Sarah A. Kopecky-Bromberg [52] 2009
					1 μg	100LD_50_ H1N1 (80%)		
			10^3^ PFU		0	100LD_50_ H1N1 (100%)		
					1 μg			
			25 PFU		0	100LD_50_ H1N1 (0%)		
					0.11 μg	100LD_50_ H1N1 (20%)		
					0.33 μg	100LD_50_ H1N1 (80%)		
					1 μg	100LD_50_ H1N1 (60%)		
					3 μg	100LD_50_ H1N1 (20%)		
mouse	intranasal	H2N2 LAIV	2 × 10^5^ PFU	IL-2	−	2 × 10^5^ PFU H2N2 (57%)	N.D.	Boris Ferko [53] 2006
					+	2 × 10^5^ PFU H2N2 (100%)		
mouse	intranasal	H1N1 LAIV	10TCID_50_	Chitosan	0	100LD_50_ H1N1 (0%)	N.D.	Ze Chen [54] 2012
					0.2%	100LD_50_ H1N1 (20%)	N.D.	
			100TCID_50_		0	100LD_50_ H1N1 (0%)	100LD_50_ H9N2 (0%)	
					0.2%	100LD_50_ H1N1 (100%)	100LD_50_ H9N2 (100%)	
			1000TCID_50_		0	100LD_50_ H1N1 (100%)	N.D.	
					0.2%	100LD_50_ H1N1 (100%)	N.D.	

^a^ N.D.: not done.

Kopecky-Bromberg *et al.* studied the application of alpha-C-GalCer as an adjuvant for an NS1‑truncated LAIV and explored its impact on vaccine effectiveness. Their results showed that co‑immunization of alpha-C-GalCer and the LAIV significantly decreased both morbidity and mortality in mice, enhanced immunogenicity of the LAIV and accelerated viral clearance in mouse lungs after challenge with a lethal dose of the wild-type virus. The presence of NKT cells was essential for adjuvant activity of alpha-C-GalCer. In addition, researchers further studied the effective dose range of alpha-C-GalCer adjuvant in immunized mice [52]. As alpha-C-GalCer is an effective adjuvant for LAIV, it certainly could also be applied to H5N1 LAIVs. 

### 3.2. Interleukin-2

Previously, many studies have demonstrated immunomodulatory cytokines could serve as adjuvant to enhance vaccine immunogenicity against a variety of infectious diseases. Interleukin-2 (IL-2) plays a key role in the stimulation and maturation process of the immune system as it stimulates the growth and differentiation of T-cells; therefore, IL-2 has often been used as a vaccine adjuvant [55]. 

The group of Boris Ferko prepared a cold-adapted LAIV where IL-2 was expressed from the NS gene and tested its immunogenicity and protective effect in young (eight-week-old) and old (18-month-old) mice. The results showed that mucosal IgA antibodies and CD8^+^ T-cells increased significantly in both young and old mice. More importantly, the IL-2 expressing cold-adapted LAIV could fully protect mice against a challenge by the wild-type influenza virus and elicit strong virus-specific CD8^+^ T-cell recall response [53]. This technology could be applied to develop a more effective H5N1 LAIV in response to a potential influenza pandemic. 

### 3.3. Chitosan

Chitosan is widely present in nature. It has a set of distinct characteristics, including non-toxic, bio‑adhesive, biodegradable, non-irritant and non-allergenic for humans; therefore, it is a safe and reliable natural active substance. It has been approved by the US FDA for use in pharmaceuticals and food [56,57]. In recent years, researchers have studied chitosan as a vaccine adjuvant, and in animal studies of vaccines for influenza, whooping cough, diphtheria, tetanus and others, chitosan has been demonstrated to enhance both systemic and local antibody responses [58,59,60]. Our laboratory has carried out in-depth studies of chitosan as an adjuvant for influenza vaccines. We found that intranasal co-immunization of mice with influenza subunit protein (M1 or sM2) and chitosan adjuvant could not only provide complete protection against challenge by homologous viruses, but also provide partial cross-protection against challenge by heterologous viruses [61,62]. We also explored whether chitosan could be applied to LAIV. We introduced mutations in PB1 and PB2 genes of influenza virus A/Puerto Rico/8/34 (H1N1), *i.e.*, PB1 (K391E, E581G, and A661T) and PB2 (N265S), and the LAIV was prepared using reverse genetics technologies. It was confirmed to have the intended features of temperature-sensitivity and attenuation. In a mouse model, immunization was performed with different doses of LAIV (10TCID_50_, 100TCID_50_ and 1000TCID_50_) adjuvanted with chitosan. Immunogenicity tests showed that chitosan adjuvant could significantly improve both humoral and cellular immune responses to the LAIV. Chitosan significantly increased influenza virus-specific IgG, IgG1 and IgG2a antibodies, in particular, the antibody titers in the 100TCID_50_ LAIV plus chitosan group was even higher than those in the 1000TCID_50_ LAIV only group, suggesting that chitosan could result in a 10‑times or higher saving in antigen dose. ELISPOT test results showed that chitosan also significantly increased the number of antigen-specific IFN-γ secreting CD8^+^ T-cells. One-time immunization of mice with the chitosan adjuvanted LAIV provided both protection against challenge by high pathogenic homologous wild-type viruses (A/Puerto Rico/8/34, H1N1) and full cross protection against challenge by heterologous virus (A/Chichen/Jiangsu/11/2002, H9N2) [54]. The chitosan adjuvant added to LAIV not only effectively reduced mice morbidity and mortality, but also expedited viral clearance in the lungs, which was indicated by virus isolation and titration experiments. The study indicated that the CD8^+^ T-cells-mediated CTL response functioned to clear influenza virus. As chitosan promoted the increase in the number of IFN-γ secreting CD8^+^ T-cells, it helped to expedite virus clearance in the lungs and, thereby, provided cross protection. Mechanisms by which chitosan enhances immune responses to LAIV is not yet clear. Chitosan might activate components of the non‑specific immune system, such as macrophages and natural killer cells; it might also decrease mucosal clearance, so that more antigens could be taken in at the nasal mucosa, thereby prolonging antigen presence in the mucosa [63]. More importantly, the cross-protective effect enhanced by chitosan adjuvant would appear particularly important when a variant of influenza virus emerges in circulation; as this cross-protection would function to clear viruses during the time when immunity in the population is still trying to catch up, thus offering better protection to humans. 

A recent randomized double-blind placebo-controlled trial showed that LAIV (FluMist^®^) immunogenicity could be partially improved by adjuvant Lactobacillus GG [64]. Although there have been no published studies about H5N1 LAIV with adjuvant, current research demonstrates that H1N1 and H2N2 LAIVs containing alpha-C-GalCer, chitosan or other novel vaccine adjuvant formulations are promising candidate vaccines and indicates that these adjuvants could be further applied to H5N1 LAIV. Developing adjuvanted H5N1 LAIV is theoretically possible, as well as feasible. Due to the “live” nature of the live-attenuated vaccines, however, during research and development of adjuvants for LAIVs, one has to consider not only characteristics of the adjuvant itself, but also has to fully take into account the environment (such as pH and ion concentration), which is required to maintain the survival of the virus.

## 4. Special Populations

In the United States, live-attenuated influenza vaccines are currently approved for use only in individuals aged 2–49 years [65]. Special populations need to receive special attention, especially in influenza outbreak periods. Studies of live-attenuated influenza vaccines in other age groups are ongoing. With the continuous development and progress of live-attenuated vaccines, it is expected that populations participating in testing of live-attenuated vaccines would continue to expand. 

### 4.1. Children

A study comparing the efficacy and safety of LAIV and TIV was carried out in children participants of 6–59 months of age (N = 7852). Compared with TIV immunized group, the LAIV immunized group had 54.9% fewer diagnosed cases of influenza (153 cases and 338 cases, *P* < 0.001). Compared to TIV, LAIV had significant protection against both antigenically well-matched and drifted viruses. Among the children of 6–11 months old, within 42 days of receiving one dose of immunization, there were 12 more cases of wheezing among the LAIV participants than the TIV participants (3.8% *vs.* 2.1%, *P* = 0.0076). Moreover, in this age group, a higher rate of hospitalization was seen in the LAIV participants than in the TIV participants (6.1% *vs.* 2.6%, *P* = 0.002). However, for the older children of 12–59 months of age, in each of the age subgroups, the hospitalization rate was lower in the LAIV participants than that in the TIV participants (*P* = 0.07). In children, the effectiveness of live-attenuated vaccine was clearly superior to the inactivated vaccine. A risks and benefits assessment indicated that for children of 12–59 months of age, live-attenuated vaccine should be a safe and highly effective vaccine [13]. For the current LAIV, it is not recommended for use in children under two years of age, children under five years of age with a history of recurrent wheezing or children with asthma, as well as adults of any age with asthma [18].

### 4.2. The Elderly

Influenza prevalence among populations over 60-year-old is a matter of public health. Increase in age brings about detrimental changes in the adapted immune system, leading to infections and suboptimal responses to preventive vaccination. In the elderly, pre-immunization antibody levels seriously hinder vaccines to function fully [66]. More and more studies have been carried out to analyze whether live-attenuated influenza vaccine is an effective strategy for the elderly in improving protection against the threat of an influenza pandemic.

A randomized, double-blinded, placebo-controlled study involving 3,242 participants with an average age of 69.5 years evaluated the efficacy, safety and immunogenicity of a live-attenuated influenza vaccine. Compared with the placebo group, LAIV was 42.3% more effective against antigenically matched influenza viruses and 52.5% more effective against heterologous viruses. Within 11 days after immunization, symptoms, including runny/stuffy nose, cough, sore throat, headache, muscle aches, tiredness and loss of appetite, occurred at a slightly higher rate in LAIV participants than in the placebo participants (*P* = 0.042), while rate of serious adverse reaction was not different between the two groups. This was the first report among populations over 60 years of age for which LAIV had significant effective protection [67].

In 2002, a randomized, open clinical study was carried out in South Africa for comparing the safety and efficacy of LAIV and TIV in adults over 60 years of age. A total of 3,009 participants were randomly divided into two groups, respectively receiving one dose of LAIV or TIV. Influenza illness caused by homologous viruses was 0.8% (12/1,494) in LAIV participants and 0.5% (8/1,488) in TIV participants. The LAIV participants tended to have fewer fevers than the TIV participants. The results on the safety of LAIV and TIV in the elderly were consistent with prior studies [68]. 

These results indicate that live-attenuated vaccines have provided one more option for the prevention of influenza in the elderly.

In sum, LAIV could provide a better protection than TIV in children and the elderly. A number of preclinical studies show that H5N1 LAIV provides good protection, and clinical trials of H5N1 LAIV are also gradually to be carried out. With the improvement of safety and efficacy for H5N1 LAIV and constant accumulation of H5N1 LAIV clinical trial data, the age range of participants about H5N1 LAIV will also expand progressively from adult to children and the elderly in the future.

## 5. Conclusions

Live-attenuated influenza vaccine could provide protection against influenza viruses of different subtypes. Multiple types of H5N1 live-attenuated vaccines could be developed based on existing vaccine production technologies and capacities. In addition, the research and development of adjuvants for live-attenuated vaccines is worthy of attention and effort, as safe and effective adjuvants can effectively lower the dose of vaccines and expand suitable ranges of vaccination. 
